# Supplementary figures and images for: A Fully-Automated Senescence Test (FAST) for the high-throughput quantification of senescence-associated markers
Source: GeroScience. 2024 Jun 13;46(5):4185–202. doi: 10.1007/s11357-024-01167-3 (PMC11336018; doi:10.1007/s11357-024-01167-3)

**a**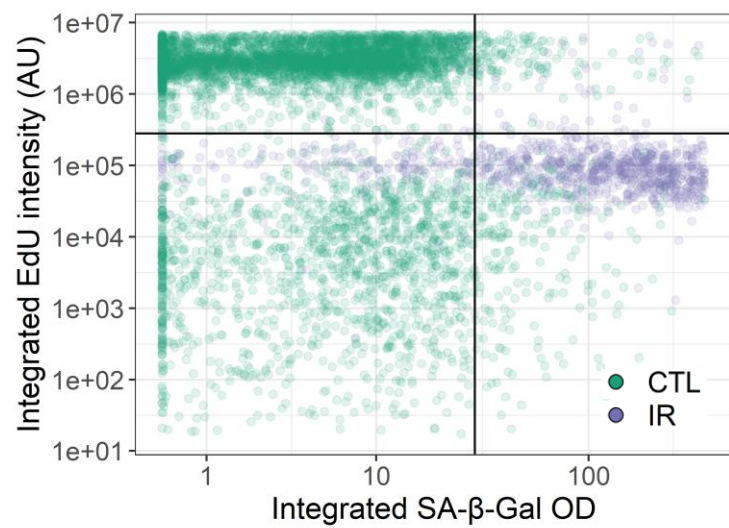**b**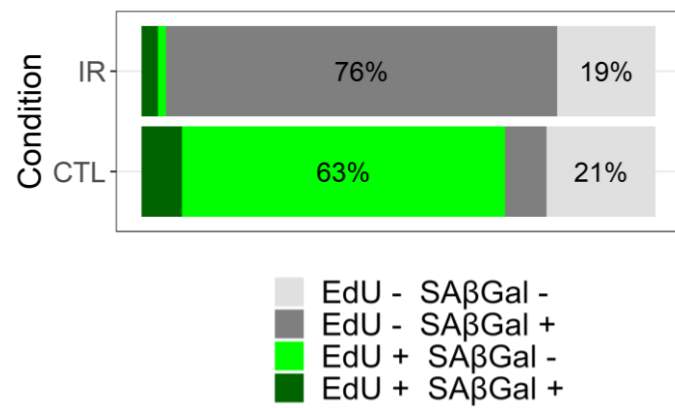**c**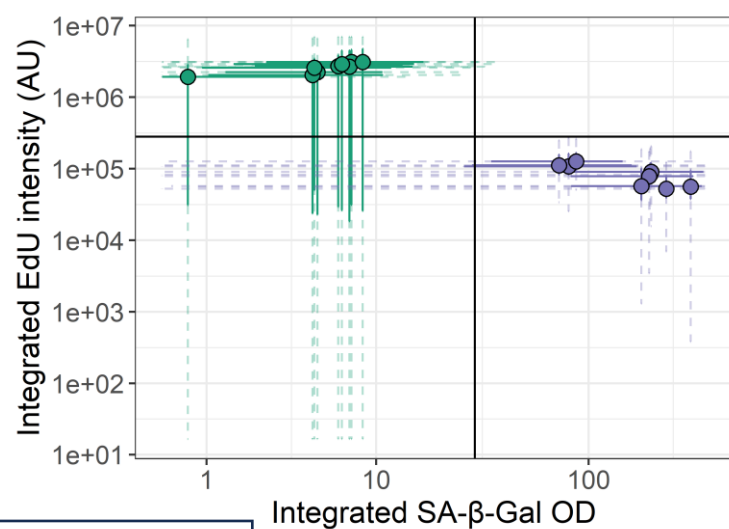**d**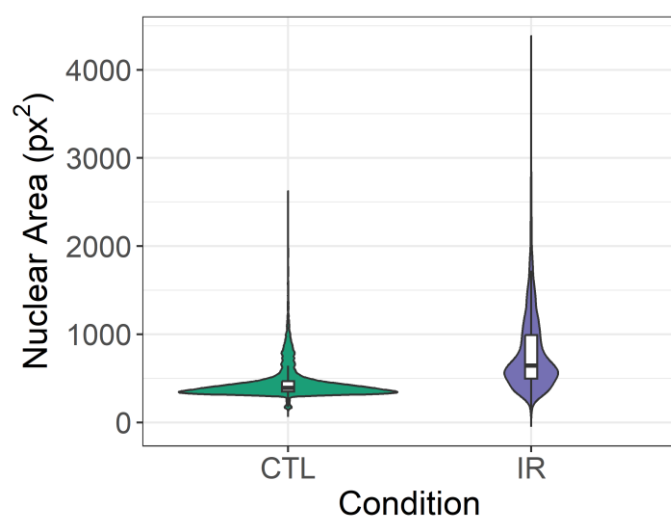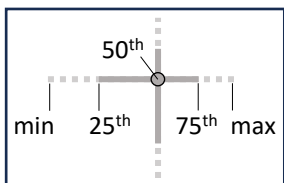

Supplement: Supplementary file 1 — Supplementary Fig. 1 Additional graphs generated with FAST. a) Scatterplot showing SA-β-Gal and EdU signal intensity of all cells. Each dot is a cell (n cells: CTL = 6359, IR = 1183). b) Bar graph showing the percentage of all cells belonging to one of the four possible staining categories: EdU+/-, SA-β-Gal +/-. c) 2D boxplot showing SA-β-Gal and EdU signal intensity of cells grouped by well. Dots indicate median (50th percentile) values, solid lines show interquartile (25th to 75th percentile) range, dashed lines show min to max range. Each data point is a well (n = 9) from the same plate. d) Violin plot showing nuclear area distribution. (PDF 230 kb) [file 11357_2024_1167_MOESM1_ESM.pdf]

**a**

Live cells

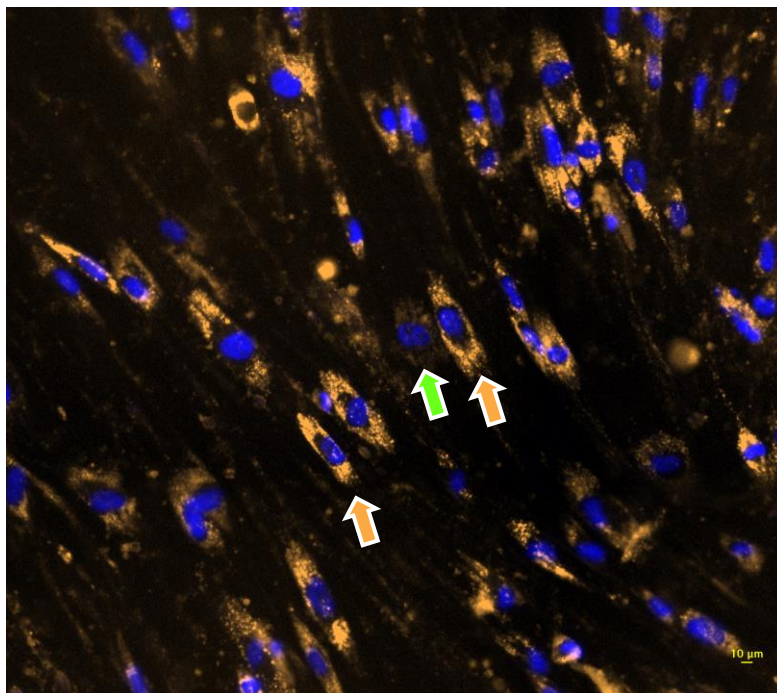

**b**

Fixed &  
permeabilized cells

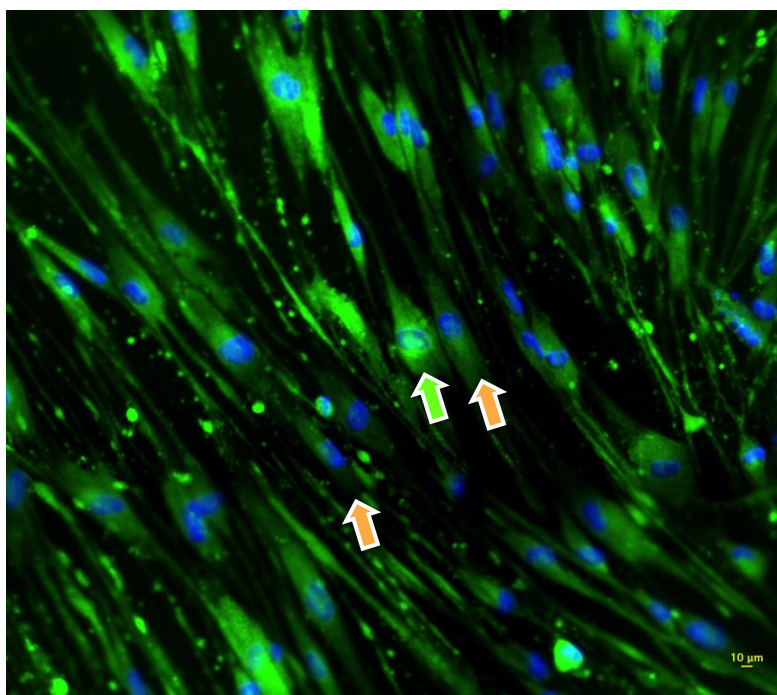

**c**

Merge

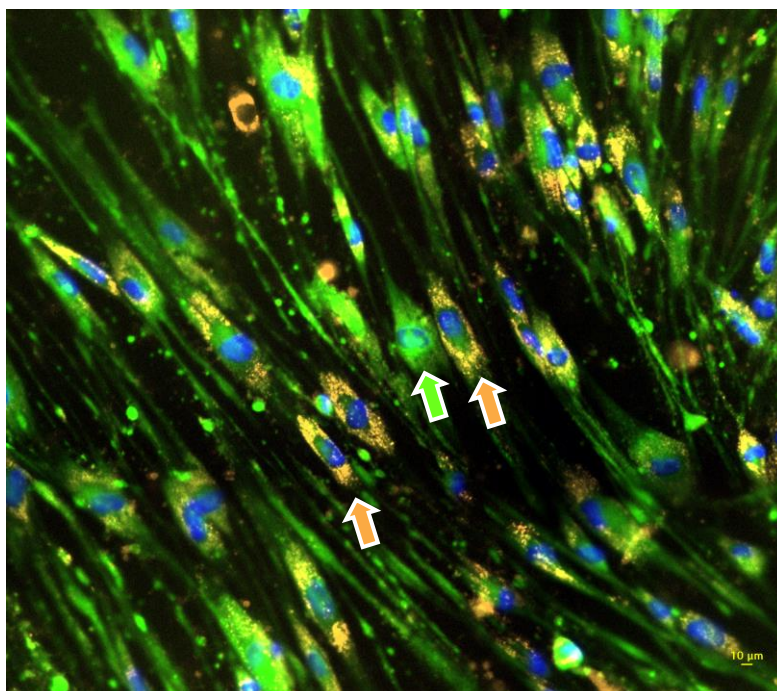

DAPI, Live, Fixed

Supplement: Supplementary file 3 — Supplementary Fig. 3 C12FDG SA-β-Gal redistributes inside and in between cells upon fixation and permeabilization. a-c) Live (a), fixed (b), and merged images (c) of senescent IMR-90 fibroblasts stained with C12FDG. For ease of distinction, fluorescence from live images is shown in orange, while fluorescence from fixed cells is shown in green. Orange arrows indicate example cells with bright staining during live imaging that is subsequently lost after fixation and permeabilization. Green arrow shows an example cell with low staining during live imaging which subsequently becomes highly fluorescent after fixation and permeabilization. (PDF 251 kb) [file 11357_2024_1167_MOESM3_ESM.pdf]

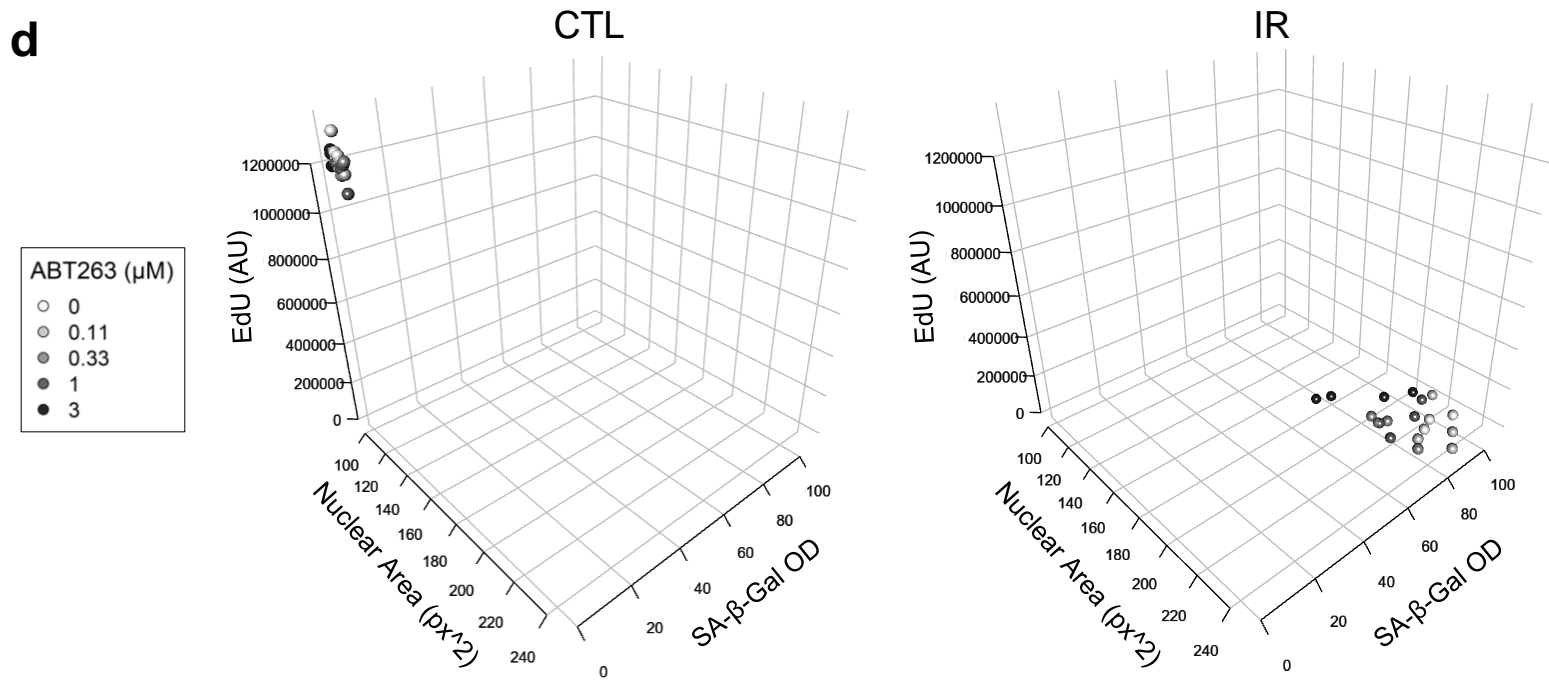

Supplement: Supplementary file 4 — Supplementary Fig. 4 ABT263 treatment does not affect SA-β-Gal and EdU staining in senescent microvascular endothelial cells. a-c) Median SA-β-Gal (a), EdU (b), and nuclear area values (c) per well at different ABT263 concentrations in non-senescent control (CTL) and IR-induced senescent (IR) cell populations (n = 4). Non-significant ANOVA p-values (p>0.05) are shown (a,b). ns, adjusted-p>0.05; *, adjusted-p<0.05, **, adjusted-p<0.01 by Tukey’s test after significant (p<0.05) one-way ANOVA. d) 3D scatterplots with all 3 variables for CTL (left) and IR (right) wells at different ABT263 concentrations. (PDF 239 kb) [file 11357_2024_1167_MOESM4_ESM.pdf]
