# Supplementary material for: A Fully-Automated Senescence Test (FAST) for the high-throughput quantification of senescence-associated markers
Source: GeroScience. 2024 Jun 13;46(5):4185–202. doi: 10.1007/s11357-024-01167-3 (PMC11336018; doi:10.1007/s11357-024-01167-3)

Image Acquisition #1

- Microscope: Nikon Eclipse Ti-PFS wide field
- Acquisition: right after staining

Image Acquisition #2

- Microscope: Zeiss LSM 980 laser scanning confocal
- Acquisition: 4 months after staining

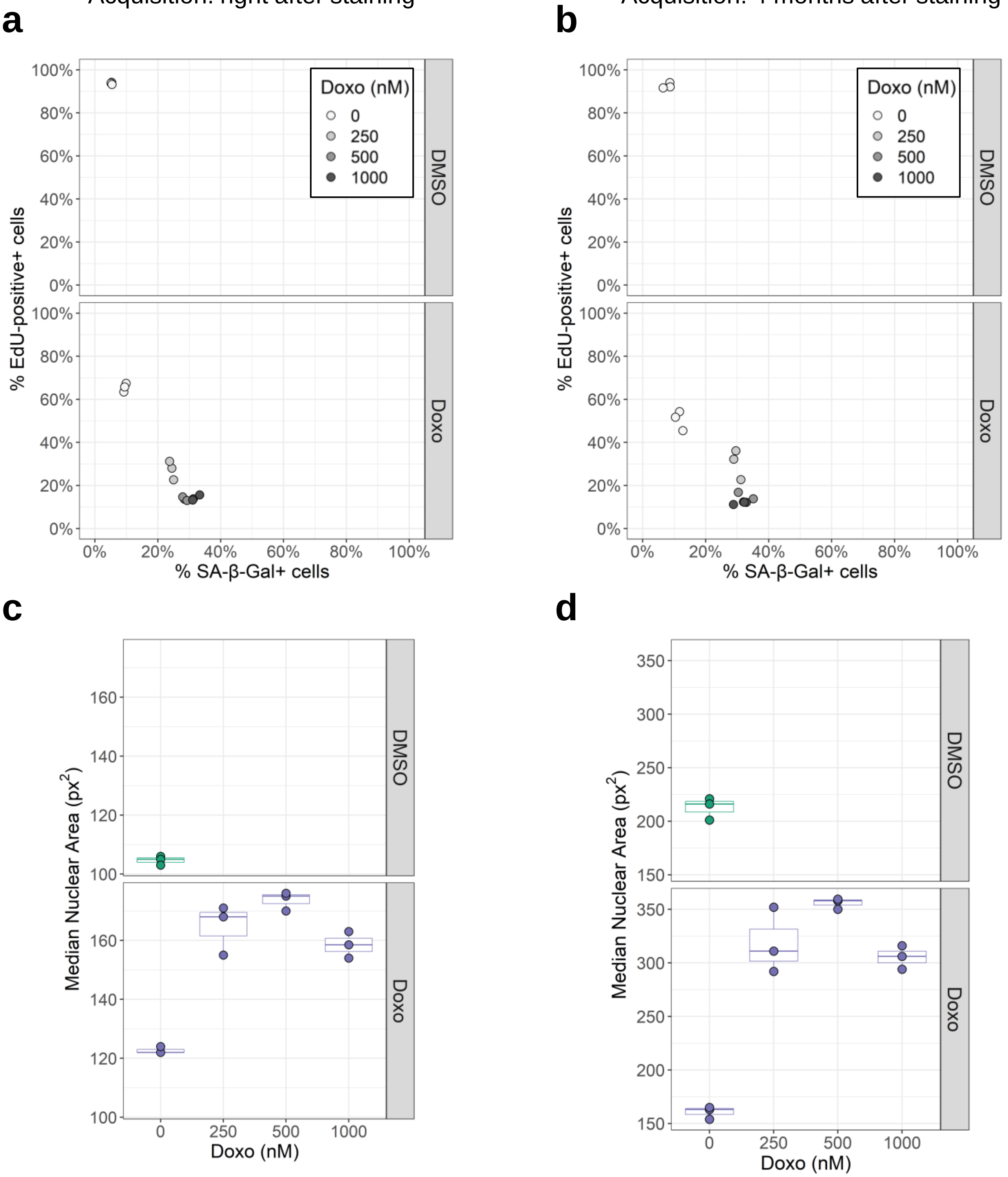

Supplement: Supplementary file 2 — Supplementary Fig. 2 FAST is compatible with different microscope setups. The same microplate containing cells treated with different concentrations of doxorubicin or DMSO vehicle was imaged with a Nikon Eclipse Ti-PFS wide-field microscope (a,c) and a Zeiss LSM 980 laser scanning confocal microscope (b,d). a,b) Percentage of SA-β-Gal- and EdU-positive cells per well for each condition. Each dot is a well (n = 4). c,d) Boxplot plot showing median nuclear area values for each condition. Each dot is a well (n = 4). (PDF 238 kb) [file 11357_2024_1167_MOESM2_ESM.pdf]
